# Supplementary material for: Water orientation and hydrogen-bond structure at the fluorite/water interface
Source: Sci Rep. 2016 Apr 12;6:24287. doi: 10.1038/srep24287 (PMC4828669; doi:10.1038/srep24287)

Water orientation and hydrogen-bond structure at the fluorite/water interface

Rémi Khatib[1,†], Ellen H. G. Backus[2,†], Mischa Bonn[2], María-José Perez-Haro[2], Marie-Pierre Gaigeot[3], and Marialore Sulpizi[1,*]

[1]Johannes Gutenberg University Mainz, Staudingerweg 7, 55099 Mainz, Germany

[2]Max Planck Institute for Polymer Research, Ackermannweg 10, 55128 Mainz, Germany

[3]LAMBE CNRS UMR8587, Université d'Evry val d'Essonne, Boulevard F. Mitterrand, Bât Maupertuis, 91025 Evry, France

[†]These authors contributed equally to this work

[*]Corresponding author: sulpizi@uni-mainz.de, +49 6131 3923641

Supplementary Figure 1: Proton distribution across the interface.

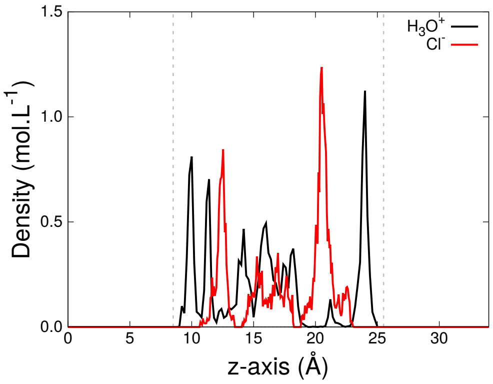

Density profile of H_3O^+ and Cl^- along the z-axis. As a guide for the eyes, the position of the CaF_2 interface is represented by a dashed grey line.

The density of hydronium (H_3O^+) and Cl^- have been plotted on Supplementary Fig. 1. The H_3O^+ has been defined as an oxygen atom surrounded by 3 hydrogens at a distance lower than 1.3 Å. One can notice that the regions with a high density of Cl^- are associated with a low density of H_3O^+ ions. This is in agreement with the low pKa of HCl and shows that HCl is fully dissociated in water.

Supplementary Figure 2: a) Schematic of the phase-resolved SFG setup. For the conventional SFG experiments the local oscillator is blocked just before the delay plate. b) Schematic of the gold reference sample and the aqueous sample cell. The SFG experiments are performed in reflection geometry at the CaF_2/gold or CaF_2/aqueous solution interface.

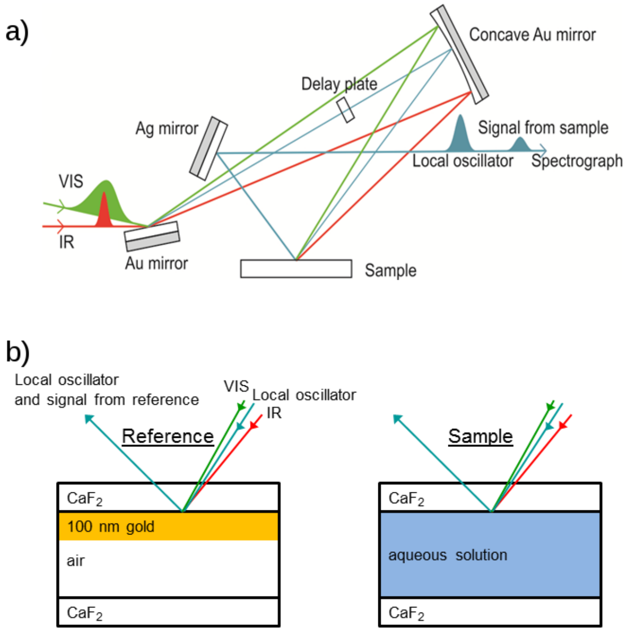

Supplementary Figure 3: Raw background corrected SFG data for the conventional a) and phase-resolved b) measurements on the gold reference sample (CaF_2/gold interface, black) and an aqueous solution of pH = 2 in contact with CaF_2 (red).

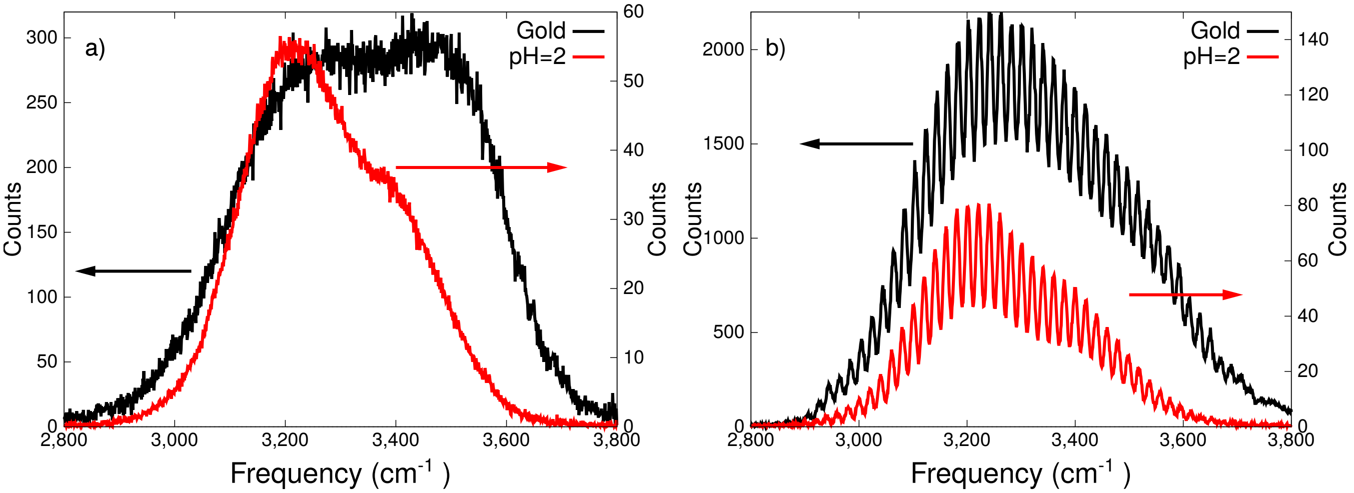

Supplement: Supplementary Information [file srep24287-s1.pdf]
